# The exposure potential restriction rule revisited

**DOI:** 10.1093/aje/kwaf204

**Published:** 2025-09-15

**Authors:** Jeremy A Labrecque, Charles Poole, Andreas Stang

**Affiliations:** Department of Epidemiology, Erasmus MC, Rotterdam, 3000 CA, The Netherlands; Department of Epidemiology, University of North Carolina at Chapel Hill, Chapel Hill, NC 27516, United States; Institute of Medical Informatics, Biometry and Epidemiology, University Hospital Essen, Essen 45147, Germany

**Keywords:** positivity, instrumental variables, confounder adjustment, bias amplification, exposure potential

## Abstract

There are people who cannot receive certain treatments or experience certain exposures. For example, people without a uterine cervix cannot receive an intrauterine device. This lack of exposure potential in some persons instigated an interesting discussion in the 1980s regarding whether such persons should be included in case–control studies. A recommendation to exclude them was named the exposure potential restriction rule. We consider this rule in the context of current modern epidemiology and causal inference including clearly defining which causal questions can be answered with which assumptions, how exposure potential relates to the positivity assumption, how the exposure potential restriction rule may amplify uncontrolled confounding when the reason for a lack of exposure potential is an instrumental variable and the complementary idea of exposure compulsion. Using a simple simulation, we demonstrate that both restricting and not restricting on a variable that defines lack of exposure may induce bias depending on the causal structure. Therefore, careful thought must be used when deciding whether to remove participants who have no potential to be exposed or no potential to be unexposed.

## Introduction

In the mid-1980′s, a discussion of whether to include people who had no ability to have been exposed in case–control studies appeared in the literature.^1^^-^^6^ A proposal to exclude them came to be known as the exposure potential restriction rule. The discussants concluded that restriction to participants with exposure potential is advisable with regard to validity only when the reason for a lack of exposure potential is a confounder. It was also added that restricting to participants with exposure potential may, in some cases, increase the precision of effect-measure estimates and, in other cases, reduce it.

Rozemeijer et al.^7^ recently revisited the exposure potential restriction rule discussion constructing an example analysis of the effect of oral contraceptives on venous thrombosis. They considered whether men, none of whom took oral contraceptives, should be included in the analysis. The inclusion of men improved the precision of the estimate of a measure of the effect of oral contraceptives on venous thrombosis when sex was not a confounder and therefore not adjusted for. When sex was a confounder and adjusting for it was required, no increase in precision was observed, except when interactions between other confounders and the exposure were present.

The aim of this manuscript is to use insights from developments in epidemiologic methods and causal inference from the past four decades to further examine the discussion of the exposure potential restriction rule. We confirm that a mere lack of exposure potential is not a sufficient reason to exclude participants. In addition, we formalize some of the assumptions required to use individuals with no exposure potential as well as consider some additional potential complications.

It should be noted that though this discussion centers around a lack of exposure potential, the same problems would occur if some groups were compelled to be exposed or, in other words, had no potential to not be exposed. The discussion below pertains equally well to no exposure potential and exposure compulsion.

## Defining exposure potential

An individual may be said to have exposure potential if they lack any characteristic or experience that entirely prevents them from being exposed. An individual with no exposure potential, on the other hand, has at least one characteristic, which entirely prevents them from being exposed. Note that exposure potential is a dichotomous characteristic, not a continuous one.

With this definition, we can define a dichotomous variable $Z$ to represent exposure potential status where $Z=1$ indicates a person with exposure potential and $Z=0$ indicates a person without exposure potential. We have chosen to code $Z$ in this way so the probability of exposure, $A,$ is zero when $Z=0$, ie, $\Pr \left[A=1|Z=0\right]=0$. For example, a person in the year 2019 could be said to have no exposure potential for receiving a COVID vaccine because the vaccines did not yet exist. Therefore, if $Z=0$ represents years before 2020 (when the first COVID vaccines were administered) and *A* is receiving a COVID vaccine, we know that the $\Pr \left[A=1|Z=0\right]=0$. Note that having exposure potential implies only that a person could be exposed ($\Pr \left[A=1|Z=1\right]>0$) not that they must be exposed $(\Pr \left[A=1|Z=1\right]=1)$. One can think about exposure compulsion in a similar way but with a $Z$ where $Z=1$ implies exposure compulsion ($\Pr \left[A=1|Z=1\right]=1$) and $Z=0$ is a lack of exposure compulsion ($\Pr \left[A=1|Z=0\right]<1$).

## When does exposure potential matter?

**Figure 1:**
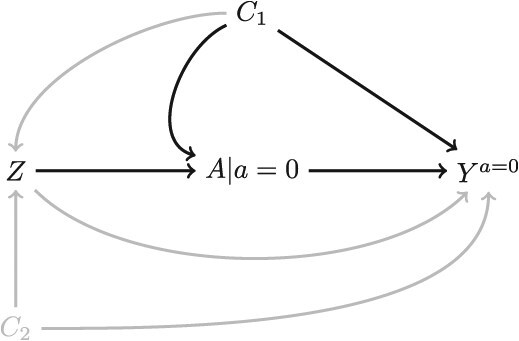
A single world intervention graph where we set a to *a* = 0 and *Y*^*a* = 0^ is a counterfactual outcome. *Z* is an indicator for having exposure potential. *C*_1_ and *C*_2_ are confounders. In order for *Z* not to be a confounder, *Z* can neither have a direct effect on ${Y}^{a=0}$ nor itself share a common cause with ${Y}^{a=0}$. If ${C}_1$ is part of the adjustment set of the main analysis, this would block the backdoor path $Z-{C}_1-{Y}^{a=0}$.

Let us begin with estimating the effect of exposure, $A$, on an outcome, $Y$, among those with exposure potential ($Z=1$). Note that this is not the treatment effect among the treated or exposed but instead the effect among those who could potentially become treated or exposed. Assuming consistency and exchangeability between $A$ and the counterfactual outcome ${Y}^a$ (possibly conditional on confounders), we can estimate this effect by simply comparing the expected value of $Y$ in the treated and untreated groups: $\mathrm{E}\left[Y|A=1,Z=1\right]-\mathrm{E}\left[Y|A=0,Z=1\right]$. When can we include data on people with no exposure potential? Including them adds observations to the $A=0$ group so instead of $\mathrm{E}\left[Y|A=0,Z=0\right]$, we would have a weighted average of the outcome between those with and without exposure potential: $\mathrm{E}\left[Y|A=0,Z=1\right]\ast P\left(Z=1|A=0\right)+\mathrm{E}\left[Y|A=0,Z=0\right]\ast P\left(Z=0|A=0\right)$. When the expected outcome under no exposure is equal in both participants with $Z=0$ and participants with $Z=1$ ($\mathrm{E}\left[{Y}^{a=0}|Z=0\right]=\mathrm{E}\left[{Y}^{a=0}|Z=1\right]$), including participants with *Z* = 0 will not harm validity because, however, they are weighted, $\mathrm{E}\left[{Y}^{a=0}\right]=\mathrm{E}\left[{Y}^{a=0}|Z=1\right]$. This assumes that the counterfactual outcome under no exposure is independent of $Z$, ${Y}^{a=0}\perp \perp Z$, an assumption of partial exchangeability. This will be satisfied when $Z$ has no effect on ${Y}^{a=0}$ except through its effect on $A$ (ie, $Z$ has no direct effect on *Y*) and there is no open backdoor path between $Z$ and ${Y}^{a=0}$. These conditions are coded in the single world intervention graph in Figure 1 as the absence of the gray arrows.^8^ If there were no open backdoor path between $A$ and $Y$, these conditions could be verified by comparing the mean value of $Y$ among unexposed participants with $Z=1$ and unexposed participants with $Z=0$. When there is an open backdoor path between $A$ and $Y$, then $\mathrm{E}\left[Y|A=0,Z=0\right]\ne \mathrm{E}\left[Y|A=0,Z=1\right]$ even when $\mathrm{E}\left[{Y}^{a=0}|Z=0\right]=\mathrm{E}\left[{Y}^{a=0}|Z=1\right]$ because conditioning on $A$ induces collider bias.

Subject matter knowledge of the data-generating mechanisms should be used to decide whether the conditions mentioned in the previous paragraph hold. For example, in a study of the effect of intrauterine devices (*A*) on depression (*Y*) that is considering including men ($Z=0$), a direct arrow from $Z$ to ${Y}^{a=0}$ may be present if being a man had a causal effect on the risk of depression when everyone had been set to not receive intrauterine devices.

**Figure 2:**
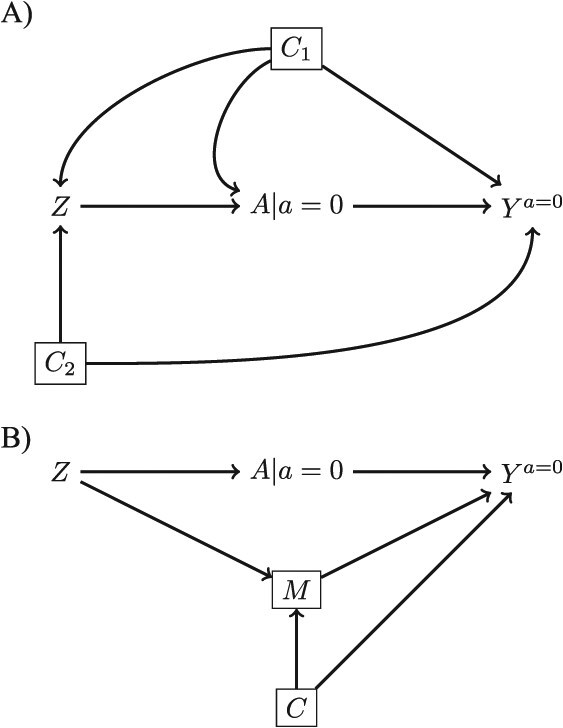
Single world intervention graphs demonstrating A) how to block confounding if the reason for no exposure potential (*Z*) is a confounder and B) how to block a direct effect of the reason for no exposure potential on the outcome.

If the conditions do not hold, there may be ways to address this problem analytically. One reason $Z$ and ${Y}^{a=0}$ might not be independent if there is one or more open backdoor paths (eg, ${C}_1$and ${C}_2$ in Figure 2A). If there are measured variables that can block these open backdoor paths then the simplest solution is to adjust for these variables. This will remove the bias caused by including people with *Z* = 0 while simultaneously potentially increasing precision.

If $Z$ itself is a cause of *Y*, the situation is more complicated. In principle, a direct effect of *Z* on ${Y}^{a=0}$ could also be blocked by conditioning on a variable on that causal path but only under two conditions: the variable is not also on a causal path from A to ${Y}^{a=0}$ and that any collider bias induced by conditioning on that variable can be controlled (Figure 2B). In practice, ensuring this would be very difficult.

## **Bias amplification when** $\boldsymbol{Z}$ **is an instrumental variable**

The example used in the original discussion of the exposure potential restriction rule only considered whether the reasons for no exposure potential, $Z$, were itself a confounder. However, whether or not there is residual confounding between the exposure and the outcome is relevant to the discussion of exposure potential restriction because of the phenomenon of bias amplification.^9^^,^^10^ When all confounding between the exposure and outcome has been controlled for and $Z$ is not itself a confounder, it is correct to argue that restricting on it will have no effect on bias but can affect the precision depending on how the restriction is accomplished. This is because a nonconfounding reason for no exposure potential is an instrumental variable: it is related to the exposure and has no association with the outcome other than by virtue of affecting the exposure. This is why many of the assumptions required in the previous section bear a noncoincidental resemblance to the assumptions required for instrumental variables.

Bias amplification occurs when bias due to residual confounding is amplified by adjusting for an instrumental variable. Therefore, the consideration of whether to exclude people with no exposure potential becomes more difficult when residual confounding may be present. If the reason for no exposure potential is an instrumental variable, then restricting on it can amplify any pre-existing bias such as that due to uncontrolled confounding. The reason for no exposure potential, $Z$, has the potential to be strongly associated with the exposure, making it a potentially strong bias amplifier, especially if the prevalence of the reason for no exposure potential is high. This greatly complicates the decision of whether one should restrict on a lack of exposure potential. If the reason for no exposure potential is a confounder, we are obliged to control for it, potentially through restriction. However, if we do control for it and it is not a confounder, we risk amplifying any existing bias (Table 1).

**Table 1:** How restriction on *Z* will affect bias in four scenarios defined by whether or not *Z* is a confounder and whether or not there is residual bias present.

	**No residual bias present**	**Residual bias present**
*Z* is a confounder	Restriction on *Z* will prevent bias caused by *Z*	Restriction on *Z* will prevent bias caused by *Z*, the residual bias will remain
*Z* is not a confounder	Restriction on *Z* will not affect bias but can decrease precision	Restriction on Z will amplify the residual bias

**Figure 3:**
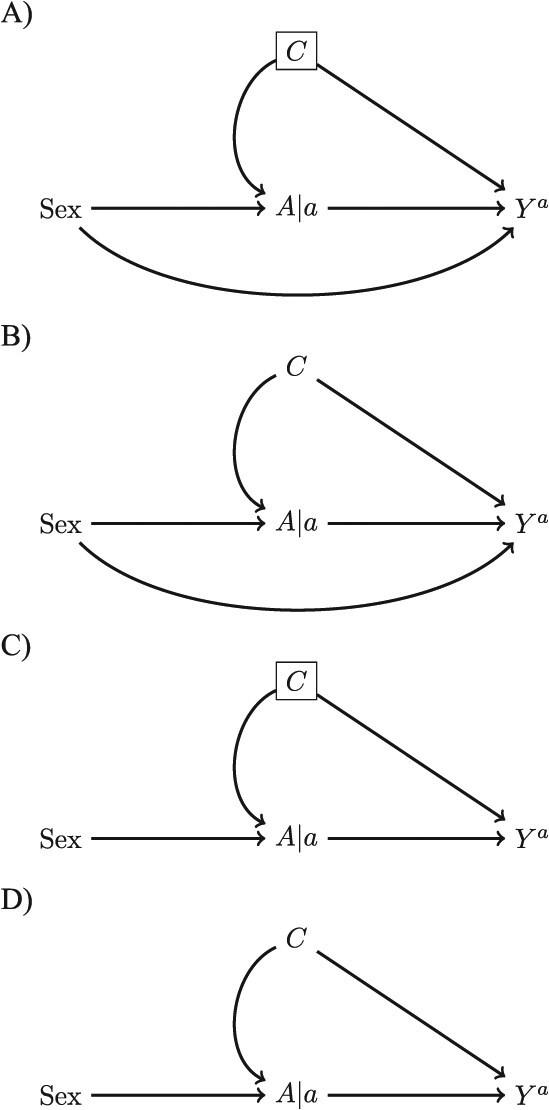
The data-generating mechanism and analyses in the simulation. Panel A) corresponds to the results rows 1-3 in Table 2, panel B) to rows 4-6, panel C) to rows 7-9, and panel D) to rows 10-12.

To illustrate this, let us use a simple simulation where we want to know the effect of binary exposure $A$ on binary outcome $Y$for which the expected estimate of a risk ratio (RR) is 1.3. Variables $A$ and $Y$ are confounded by $C$, for which the effect on both $A$ and $Y$ is RR = 1.75. $C$ does not modify the effect of $A$ on $Y$ on the RR scale. Log-binomial regression is used to estimate the effect of $A$ on $Y$ and adjustment is carried out by including the variable as a covariate. It so happens that men cannot be exposed to $A$—ie, they have no exposure potential. In the simulation, 69% of women are exposed. We investigate two data-generating mechanisms. In the first, the reason for no exposure potential, sex, is also independently preventive of the outcome (RR = 0.75). In the second, sex is not an independent risk factor for the outcome (ie, It is an instrumental variable and therefore its direct relationship with *Y* is RR = 1.) For each analysis, we used a sample size of 10 000 (5000 on average when men were excluded from the analysis) and ran 1000 iterations. In Table 2, we present the RR, bias and mean squared error. Figure 3 shows the single world intervention graph corresponding to the different data-generating processes and adjustment variables in each analysis. The code to run the simulation can be found in Appendix S1 and at https://github.com/jalabrecque/EPRR_sim.

**Table 2:** Results from simulations illustrating how adjusting for or conditioning on sex where men have no exposure potential affects the bias when estimating the effect of exposure *a*.

**Row**	**Analysis**	**Log(RR) of the effect of *A***	**SE**	**Bias**	**MSE**	**RR**
Sex as a confounder						
1	Adjusted for *C*	0.50	0.03	0.23	0.055	1.64 (1.56-1.73)
2	Adjusted for *C*, sex	0.26	0.05	0.00	0.002	1.30 (1.19-1.42)
3	Adjusted for *C*, restricted on sex	0.26	0.05	0.00	0.002	1.30 (1.19-1.43)
4	No adjustment	0.60	0.03	0.33	0.111	1.81 (1.72-1.91)
5	Adjusted for sex	0.51	0.04	0.25	0.064	1.67 (1.53-1.82)
6	Restriction on sex	0.51	0.04	0.25	0.064	1.67 (1.53-1.82)
Sex as an instrumental variable						
7	Adjusted for *C*	0.26	0.03	0.00	0.001	1.30 (1.24-1.36)
8	Adjusted for *C*, sex	0.26	0.04	0.00	0.002	1.30 (1.19-1.42)
9	Adjusted for *C*, restricted on sex	0.26	0.05	0.00	0.003	1.30 (1.19-1.42)
10	No adjustment	0.37	0.03	0.11	0.012	1.45 (1.38-1.53)
11	Adjusted for sex	0.51	0.04	0.25	0.066	1.67 (1.53-1.82)
12	Restriction on sex	0.51	0.04	0.25	0.066	1.67 (1.53-1.82)

Abbreviations: RR, risk ratio, SE, standard error; MSE, mean squared error.

The true RR is set to 1.3 (log(RR) = 0.26). The sample size for each run was 10 000, and the simulation was run 1000 times. When restricted on sex, the sample size was, on average, 5000.

When sex is a confounder, adjusting for it or restricting on it (ie, removing men from the sample) decreases bias and increases the standard error both in the presence (Figure 3A) and absence (Figure 3B) of residual confounding (rows 1–6). When sex is an instrumental variable, but there is no residual confounding (Figure 3C), adjusting for or restricting on sex does not change the bias, but it does increase the standard error (rows 8-9 versus row 7). When sex is an instrumental variable and there is residual confounding (Figure 3D), adjusting for or restricting on sex increases the standard error, as before, but also increases the bias (rows 11-12 versus row 10). Therefore, the decision on whether to restrict on a variable that is a reason for nonexposure can not only increase standard errors but can also increase bias when it is not an independent risk factor for the outcome (Table 2).

## Exposure potential and positivity

It may be tempting to think that a person with no exposure potential must necessarily violate the positivity assumption because in the stratum $Z=0$, there are only unexposed people. For the purposes of controlling for confounding, however, the model or analysis producing the propensity score should include only variables that are needed to control confounding or collider bias. If the reason for being unable to be exposed, $Z$, is itself such as variable, then $Z$ must be included in the propensity score model and therefore anyone with $Z=0$ should have a propensity score equal to 0. If conditioning on $Z$ is not required to control confounding, it should not be included in the propensity score model and therefore people with $Z=0$ need not have a propensity score equal to 0 if there are also people with $Z=1$ who are exposed in each stratum of confounders. Therefore, $Z=0$ only implies the propensity score is zero when $Z$ is a confounder and positivity need not be violated when $Z$ is not a confounder.

It is the target population, as opposed to what Maldonado and Greenland^11^ have called “substitute populations” for exposure or treatment conditions the target population has not factually experienced, who must be composed only of individuals who are able to experience both exposure levels. In other words, target population “positivity”^11^ requires that everyone for whom a causal effect measure is being estimated must have exposure potential and also potential to not be exposed. The only valid target population, therefore, is all individuals with $Z=1$ who are able to be exposed and able to be not exposed (ie, people with exposure potential and who are not compelled to exposure). It should be noted, however, that when there is no open causal or noncausal path from $Z$ to ${Y}^a$, $Z$ cannot modify the effect of $A$ on $Y$ and therefore the effect in the $Z=1$ stratum will be equal to the effect in the $Z=0$ stratum.^12^

There are various ways of dealing with positivity violations including restriction, interpolation, extrapolation, not adjusting for the confounders responsible for the positivity violation and modifying the causal question.^13^ In most cases, groups with no exposure potential will be defined by a categorical variable (eg, severe CKD yes versus no) and interpolation and extrapolation will not work. Restriction is one of the simplest ways of addressing positivity issues although at the cost of a potential loss of precision. Not adjusting for the variable is another simple solution though this will incur bias to the degree that *Z* is a confounder or is a collider on an otherwise open path. One additional way is to restrict the inference to only the group where positivity is satisfied (ie, persons without severe CKD) while keeping all subjects in the statistical model. This is done by creating a categorical variable with all possible combinations of exposure and the variable where positivity is not satisfied. Rozemeijer et al.^7^ illustrated that adding the exposure of interest and the reason for no exposure potential as independent variables in a logistic model “yields a model equivalent to adding a categorical covariate with 3 categories”: unexposed subjects without exposure potential, exposed subjects with exposure potential, and unexposed subjects with exposure potential. In other words, the model will estimate the average outcome in each of the categories separately and no information from the group with no exposure potential will be used to estimate the effect in those with exposure potential. The group with no exposure potential only aids in estimating other, nuisance parameters in the model (ie, the coefficients of covariates).

## Reasons for a lack of exposure potential

In the process of coming up with examples for this manuscript, we discovered that it is, in fact, not easy to come up with situations where a person would truly have absolutely zero exposure potential. In many cases in which a person might be considered to have no exposure potential, one can almost inevitably come up with a way, perhaps farfetched, in which such a person could become exposed. Some medical treatments may be strongly contraindicated for people with specific conditions (eg, the absolute contraindication for clozophine, which may cause severely low white blood cell counts, in people with a history of bone marrow suppression), yet it remains physically possible for such people to receive those treatments. Thus, even an absolute contraindication, which means only that there is no reasonable circumstance under which a person with a specific condition should take the treatment in question, does not make it absolutely impossible that they could receive it. In a similar vein, Rozemeijer et al.^7^ discussed men as having no exposure potential for oral contraceptive use, yet persons who might be classified as men by one criterion or another can and do take oral contraceptives on occasion. Therefore, in practice, very often, “no exposure potential” operationally tends to indicate the presence of circumstances that render receipt of the exposure extraordinarily unlikely.

Of the many different reasons an individual may have no exposure potential, we will discuss three for illustrative purposes. The first is that the exposure is not well defined in the group with no exposure potential. For example, although it can be written in words, there is no meaningful interpretation of “giving a person without a uterine cervix an intrauterine device” as such a person would have no uterus in which to place the device. Therefore, if $Z=0$ indicates a person without a uterine cervix and $A=1$ indicates administering an intrauterine device, the potential outcome $\Pr \left[{Y}^{a=1}|Z=0\right]$ is not well defined. It should be noted that the potential outcome under no exposure, Pr$\left[{Y}^{a=0}\mid Z=0\right]$, remains well defined.

A second reason is absolute contraindication. If $Z=0$ represents a reason for absolute contraindication for a treatment, the counterfactual outcome under treatment is well defined in people with $Z=0$ but is never, or almost never, observed either because exposure would lead to death or adverse health outcomes, as was the case in the clozophine example given earlier.

A third reason for lack of exposure potential may simply be that an exposure (or treatment) did not exist yet when the data were collected. Prior to 2020, every person on Earth had no exposure potential for any COVID vaccine because they had not yet been developed. Today, on the other hand, most people, no matter how remotely located on the planet, will have at least some exposure potential for COVID vaccines.

This last reason for a lack of exposure potential is worth exploring. In analyses of small randomized trials of new treatments, data from people who could not have had access to the treatment (and thus had no exposure potential), sometimes called historical controls, can be included to help in the estimation of the mean outcome in the control group of the trial.^14^ When using historical controls, a lack of exposure potential is used as a rationale for inclusion rather than a rationale for exclusion. This is because a lack of exposure potential in a population means that there is no decision regarding whether to be treated and therefore there can be no confounding resulting from treatment decisions being made based on risk factors for the outcome. In Figure 1, this means that confounders such as ${C}_1$, with an arrow pointing into treatment $A$, cannot exist. Confounding in studies including historical controls stems from potential unblocked backdoor paths through $Z$ which, in this case, is an indicator variable for being in the trial data versus being in the historical control data. For example, if trial participants ($Z=1$) in the control arm ($A=0$) receive better care or are somehow treated differently, simply by virtue of being enrolled in the trial, than the care received by historical controls, then participation in the trial, $Z$, may have an effect on the outcome that is not due to the exposure (the direct arrow from $Z$ to ${Y}^a$ in Figure 1). $Z$ can also be confounded with $Y$, if, the historical controls were sampled at a different time than the trial and there was a secular trend in the outcome. This would be similar to variable ${C}_2$ in Figure 1.

The rationale for the inclusion of individuals with no exposure potential in a more general context should follow the same logic as that for the use of historical controls. That is, individuals with no exposure potential can aid in the estimation of the average outcome among the unexposed with exposure potential unless their presence results in confounding that cannot be addressed or reduces precision appreciably.

## Conclusion

Viewing the exposure potential restriction rule in light of modern epidemiologic methods and causal inference provides important insight. One of the original postulated reasons for the rule was that it ensures focus on questions of relevance.^2^ We demonstrate that the relevant target population is the same regardless of restriction and that people with no exposure potential can help estimate the counterfactual outcome under no exposure if the reason for no exposure potential is either not a confounder or that the confounding can be controlled. Additionally, restriction to individuals with exposure potential can amplify any residual bias when the reason for no exposure potential is an instrumental variable. The exposure potential restriction rule is therefore not necessary and may, in some cases, increase bias.

## Supplementary Material

Web_Material_kwaf204

## Data Availability

No data were used in this manuscript.
