# Supplementary material for: The exposure potential restriction rule revisited
Source: Am J Epidemiol. 2025 Sep 15;194(12):3624–9. doi: 10.1093/aje/kwaf204 (PMC12671978; doi:10.1093/aje/kwaf204)
Supplement: Web_Material_kwaf204 [file web_material_kwaf204.zip › EPRR_Supplemental_material_FINAL.docx]

**Supplementary Data**

**Article:** The exposure potential restriction rule revisited

Authors: Jeremy A. Labrecque, Charles Poole, Andreas Stang

Contents:

Appendix S1: Code for simulation…………………………………………………………………………………..2

**Code for simulation**

The code can also be found at xxxx

**Parameters and code for the simulation:**

n = 10,000

Effect of C on A: RR=1.75

Effect of C on Y: RR=1.75

Effect of A on Y: RR=1.3

Effect of Sex on Y: RR=0.75

**Code:**

# The only required library is 'magrittr'

# install.packages("magrittr")

# The following functions have the parameters from the manuscript as their

# defaults but these defaults are easily changed in the inputs.

# The simulation is generated on the log scale, not the logit scale, so it is

# possible for the some parameters to not work because they lead to probabilities

# greater than one.

# This function runs one simulation and analyzes it

eprr_sim <- function(n = 10000, c_a = 1.75, c_y = 1.75, a_y = 1.3, sex_y = 0.75) {

# SEX AS A CONFOUNDER

## Generate data

sex <- rbinom(n = n, size = 1, prob = 0.5)

c <- rbinom(n = n, size = 1, prob = 0.5)

a <- rbinom(n = n, size = 1, prob = exp(log(0.33/0.66) + log(c_a)*c))

a[sex==1] <- 0 # Set the exposure in men to 0

y <- rbinom(n = n, size = 1, prob = exp(log(0.2/0.8) + log(a_y)*a + log(c_y)*c + log(sex_y)*sex))

ds_c <- data.frame(sex, c, a, y)

## Adjusting for C

mod_corr <- glm(y ~ a + c, data = ds_c, family = binomial(link=log))

est_corr <- summary(mod_corr)$coefficients["a",c("Estimate","Std. Error")]

mod_corr_adj_sex <- glm(y ~ a + c + sex, data = ds_c, family = binomial(link=log))

est_corr_adj_sex <- summary(mod_corr_adj_sex)$coefficients["a",c("Estimate","Std. Error")]

mod_corr_restr_sex <- glm(y ~ a + c, data = ds_c[ds_c$sex==0,], family = binomial(link=log))

est_corr_restr_sex <- summary(mod_corr_restr_sex)$coefficients["a",c("Estimate","Std. Error")]

## Not adjusting for C

mod_biased <- glm(y ~ a, data = ds_c, family = binomial(link=log))

est_biased <- summary(mod_biased)$coefficients["a",c("Estimate","Std. Error")]

mod_biased_adj_sex <- glm(y ~ a + sex, data = ds_c, family = binomial(link=log))

est_biased_adj_sex <- summary(mod_biased_adj_sex)$coefficients["a",c("Estimate","Std. Error")]

mod_biased_restr_sex <- glm(y ~ a, data = ds_c[ds_c$sex==0,], family = binomial(link=log))

est_biased_restr_sex <- summary(mod_biased_restr_sex)$coefficients["a",c("Estimate","Std. Error")]

res <- as.data.frame(rbind(est_corr, est_corr_adj_sex, est_corr_restr_sex,

est_biased, est_biased_adj_sex, est_biased_restr_sex))

res$bias <- res$Estimate - log(a_y)

out_c <- c(t(res))

names(out_c) <- paste0("c_",apply(expand.grid(c("est","se","bias"), c("corr","corr_adj_sex","corr_restr_sex", "biased","biased_adj_sex","biased_restr_sex")), 1, paste, collapse="_"))

# SEX AS AN IV

## Data generation

sex <- rbinom(n = n, size = 1, prob = 0.5)

c <- rbinom(n = n, size = 1, prob = 0.5)

a <- rbinom(n = n, size = 1, prob = exp(log(0.33/0.66) + log(c_a)*c))

a[sex==1] <- 0

y <- rbinom(n = n, size = 1, prob = exp(log(0.175/0.8) + log(a_y)*a + log(c_y)*c)) ; mean(y)

ds_iv <- data.frame(sex, c, a, y)

## Adjusting for C

mod_corr <- glm(y ~ a + c, data = ds_iv, family = binomial(link=log))

est_corr <- summary(mod_corr)$coefficients["a",c("Estimate","Std. Error")]

mod_corr_adj_sex <- glm(y ~ a + c + sex, data = ds_iv, family = binomial(link=log))

est_corr_adj_sex <- summary(mod_corr_adj_sex)$coefficients["a",c("Estimate","Std. Error")]

mod_corr_restr_sex <- glm(y ~ a + c, data = ds_iv[ds_iv$sex==0,], family = binomial(link=log))

est_corr_restr_sex <- summary(mod_corr_restr_sex)$coefficients["a",c("Estimate","Std. Error")]

## Not adjusting for C

mod_biased <- glm(y ~ a, data = ds_iv, family = binomial(link=log))

est_biased <- summary(mod_biased)$coefficients["a",c("Estimate","Std. Error")]

mod_biased_adj_sex <- glm(y ~ a + sex, data = ds_iv, family = binomial(link=log))

est_biased_adj_sex <- summary(mod_biased_adj_sex)$coefficients["a",c("Estimate","Std. Error")]

mod_biased_restr_sex <- glm(y ~ a, data = ds_iv[ds_iv$sex==0,], family = binomial(link=log))

est_biased_restr_sex <- summary(mod_biased_restr_sex)$coefficients["a",c("Estimate","Std. Error")]

res <- as.data.frame(rbind(est_corr, est_corr_adj_sex, est_corr_restr_sex,

est_biased, est_biased_adj_sex, est_biased_restr_sex))

res$bias <- res$Estimate - log(a_y)

out_iv <- c(t(res))

names(out_iv) <- paste0("iv_",apply(expand.grid(c("est","se","bias"), c("corr","corr_adj_sex","corr_restr_sex", "biased","biased_adj_sex","biased_restr_sex")), 1, paste, collapse="_"))

return(c(out_c, out_iv))

}

# This function replicates the eprr_sim function 'iter' number of times

eprr_replicate <- function(iter = 10, n = 10000, c_a = 1.75, c_y = 1.75, a_y = 1.3, sex_y = 0.75, r=3) {

library(magrittr)

reps <- replicate(iter,eprr_sim(n = n, c_a = c_a, c_y = c_y, a_y = a_y, sex_y=sex_y))

results <- rowMeans(reps) %>% matrix(.,nrow=12, byrow = TRUE) %>%

as.data.frame %>%

`row.names<-`(c("c_corr","c_corr_adj_sex","c_corr_restr_sex", "c_biased","c_biased_adj_sex","c_biased_restr_sex","iv_corr","iv_corr_adj_sex","iv_corr_restr_sex", "iv_biased","iv_biased_adj_sex","iv_biased_restr_sex")) %>%

`names<-`(c("est","se","bias"))

results$MSE <- rowMeans(reps[grep(row.names(reps),pattern = "bias_"),]^2)

results$RR <- exp(results$est)

results$ci_low <- exp(results$est - qnorm(0.975)*results$se)

results$ci_high <- exp(results$est + qnorm(0.975)*results$se)

return(round(results,r))

}

# Runs the simulation in the paper

ans <- eprr_replicate(iter=1000)

# Add a character string with the RR and CI

ans$RR_CI <- paste0(format(round(ans$RR,2),nsmall=2),

" (",

format(round(ans$ci_low,2),nsmall=2),

"-",

format(round(ans$ci_high,2),nsmall=2),

")")
